# DNMT gene expression in peripheral leukocytes in schizophrenia and correlations with one-carbon metabolites: folate, total homocysteine, and vitamin B6

**DOI:** 10.3389/fpsyt.2025.1715372

**Published:** 2026-01-19

**Authors:** Yukiko Tomioka, Masahito Nakataki, Hidehiro Umehara, Tomohiro Yoshida, Hiroya Matsuda, Yui Matsumoto, Mariko Aoki, Yuri Yoshida, Yuichiro Kamiyama, Tomohiko Nakayama, Naoki Yamada, Shusuke Numata

**Affiliations:** 1 Department of Psychiatry, Graduate School of Biomedical Science, Tokushima University, Tokushima, Japan; 2 Accessibility Division, Health Service, Counseling and Accessibility Center, Tokushima University, Tokushima, Japan

**Keywords:** DNA methylation, DNA methyltransferase (DNMT), folate, gene expression, schizophrenia, vitamin B6

## Abstract

**Aims:**

Previous studies have identified alterations in one-carbon metabolism (OCM), including DNA methylation abnormalities, in individuals with schizophrenia. However, the precise etiology of this disorder remains unclear. In the present study, we examined variations in the expression of *DNMT1 and DNMT3a*—genes implicated in DNA methylation—using peripheral blood leukocytes from Japanese patients with chronic schizophrenia and healthy controls. Additionally, using our previously acquired data, we explored the association between OCM-related factors and *DNMT* expression levels.

**Methods:**

Expression levels of *DNMT1* and *DNMT3a* in 215 patients with chronic schizophrenia and 210 healthy controls were quantified using real-time PCR. The Mann-Whitney U test was used to compare the differences between two independent groups. Furthermore, Spearman’s correlation analysis was conducted to investigate the relationships between *DNMT* genes` expression levels and OCM-related metabolites (blood folate, vitamin B6, and total homocysteine).

**Results:**

The expression levels of *DNMT1* and *DNMT3a* in peripheral leukocytes were significantly elevated in patients with chronic schizophrenia compared with controls (p = 1.4 × 10–^6^ and 2.9 × 10^-3^, respectively). DNMT1 mRNA expression levels exhibited a weak negative correlation with folate exclusively in the aggregated cohort (N = 425) (ρ = −0.16, adjusted q = 5.0 × 10^-3^), and DNMT3a mRNA expression levels showed a weak negative correlation with vitamin B6 alone in the combined group (ρ = −0.12, adjusted q = 0.03).

**Conclusion:**

These findings suggest a potential correlation between nutritional status and elevated expression of DNMT1 and DNMT3a in schizophrenia. Our findings contribute to the understanding of the epigenetic mechanisms associated with schizophrenia and highlight the need for further investigation of the relationships among gene expression, nutritional status, and psychiatric manifestations.

## Introduction

Schizophrenia is a psychotic disorder characterized by positive symptoms, such as hallucinations and delusions, and negative symptoms, such as decreased motivation and cognitive dysfunction. The disorder has a lifetime prevalence of approximately 0.7–0.8% and typically manifests during adolescence and young adulthood, following a chronic course. However, the etiology of the disease is not yet fully understood.

One-carbon metabolism (OCM) involves the transfers of one-carbon groups by folate to a variety of biological processes, including DNA synthesis, DNA methylation, and homocysteine metabolism. It has been indicated that the OCM may play an important role in the pathophysiology of schizophrenia (1, 2). Our work focused on one-carbon metabolism and demonstrated elevated plasma total homocysteine (tHcy) levels, decreased serum pyridoxal (a form of vitamin B6) and folate levels, and DNA methylation abnormalities in schizophrenia (3–7). Furthermore, we have reported associations between tHcy and DNA methylation at specific genes as well as correlations of serum folate with plasma tHcy and serum pyridoxal levels (5, 8). Within OCM, DNA methyltransferases (DNMTs) catalyze DNA methylation, an essential epigenetic process. DNMT genes include *DNMT1*, responsible for maintenance of methylation, and *DNMT3a* and *DNMT3b*, which mediate *de novo* methylation. Altered gene expression and protein expression of DNMTs has been reported in the peripheral blood and brain tissues of patients with schizophrenia (9–16). However, these studies have generally involved small sample sizes, and no studies to date have systematically examined the association between DNMT gene expression and OCM-related metabolites.

In this study, we first investigated DNMT gene expression in the peripheral leukocytes of patients with schizophrenia, followed by an analysis of the relationships between the DNMT gene expression and metabolites within OCM.

## Method

### Participants

A total of 215 patients with chronic schizophrenia (108 males, mean age 51.06 ± 9.30 years; 107 females, mean age 55.52 ± 9.60 years) were recruited from Tokushima University Hospital and affiliated psychiatric hospitals between October 2011 and March 2014. Schizophrenia was diagnosed by at least two experienced psychiatrists based on the DSM-IV criteria, comprehensive clinical interviews, and review of medical records (**Table 1**), as patient recruitment was initiated prior to the publication of DSM-5 and diagnostic criteria for schizophrenia are largely comparable between DSM-IV and DSM-5.

**Table 1:** Demographic and biochemical characteristics of participants.

Variable	Control	Chronic schizophrenia	p-value
N	210	215	
Sex (Male/Female)	106/104	108/107	1
Age	53.20 ± 9.34	53.28 ± 9.69	0.79
Male mean age	50.25 ± 8.87	51.06 ± 9.30	0.42
Female mean age	56.21 ± 8.88	55.52 ± 9.60	0.86
median CP (min-max)	–	725 (0–2650)	–
folate	6.10 ± 2.61	3.78 ± 1.87	2.0 × 10^-27^
vitamin B6	12.78 ± 11.47	6.12 ± 5.87	3.1 × 10^-32^
tHcy	12.47 ± 5.13	19.22 ± 14.79	6.2 × 10^-18^

Almost all patients were hospitalized and treated with various antipsychotic medications. The median chlorpromazine-equivalent dose was 725 mg/day (range, 0–2650 mg/day). Chlorpromazine-equivalent doses were calculated according to previously published conversion tables (Inada and Inagaki, 2015) (17). In patients receiving antipsychotic polypharmacy, total daily chlorpromazine-equivalent doses were calculated by summing the equivalents of each antipsychotic medication. Non-psychiatric controls were recruited from hospital staff, students, and company employees; structured interviews were not included for controls. The control group was matched to the schizophrenia group by age and sex. The study was approved by the Ethics Committee of Tokushima University Hospital, and all participants provided written informed consent. The study was conducted in accordance with the Declaration of Helsinki.

### Tissue processing, RNA purification, and sample preparation for real-time PCR analysis

Whole blood samples were collected, and total RNA was extracted using PAXgene Blood RNA tubes (Qiagen N.V., Hilden, Düsseldorf, Germany) and the PAXgene Blood RNA kit (Qiagen) following the manufacturer’s instructions. RNA quality was determined using an Agilent 2100 Bioanalyzer with LabChip technology (Agilent Technologies, Santa Clara, CA, USA). The cutoff value for RNA integrity was set at 5.0. In this dataset, the lowest RNA integrity number (RIN) was 5.1, and the mean RIN was 8.5 ± 0.7. RNA quantification was performed using a NanoDrop ND-1000 spectrophotometer (Thermo Fisher Scientific Inc., Waltham, Massachusetts, USA). After assessing the quality and quantity of RNA, 250 ng of each total RNA sample was reverse transcribed into cDNA using the QuantiTect Reverse Transcription Kit (Qiagen).

### DNMTs expression by real-time PCR

DNMT mRNA expression levels were measured in triplicate using quantitative real-time PCR on an Applied Biosystems StepOnePlus Real-Time PCR System (Thermo Fisher Scientific). The ΔΔCt method was used to determine relative expression levels in schizophrenia and control samples. *ACTB* was used as a reference gene. The following probes were used: *DNMT1* - Hs.PT.58.28037916; *DNMT3a* - Hs.PT.58.4543699; *ACTB* and ACTB-Hs.PT.39a.22214847 (Integrated DNA Technologies).

### Measurement of folate, vitamin B6, and homocysteine

Serum pyridoxal and plasma total homocysteine (tHcy) levels were measured using high-performance liquid chromatography (HPLC). Serum folate concentrations were measured by chemiluminescent enzyme immunoassay using the Access Folate Assay Kit (Beckman Coulter, Brea, CA, USA). Serum pyridoxal levels were measurable above 2.0 ng/mL, and serum folate concentrations ranged from 4.0 to 20.0 ng/mL. All samples were analyzed at the SRL Laboratory (Tokyo, Japan). The participants in the present study overlapped with those of our previous studies (3–5) and blood data for the overlapping samples were obtained from these studies. DNMT gene expression and biochemical measurements, including folate, vitamin B6, and total homocysteine, were obtained from the same peripheral blood samples collected at the time of study participation.

### Statistical analysis

Demographic differences in age between patients with schizophrenia and controls were compared using the Mann-Whitney U test. Sex differences between the two groups were assessed using chi-square tests. The Mann-Whitney U test was also used to compare continuous variables between independent samples. Spearman’s correlation analysis was conducted to examine the relationships between DNMT gene expression and OCM-related metabolites. All statistical analyses were performed using R software (version 4.3.1), with statistical significance set at p = 0.05. A false discovery rate (FDR) approach was applied to adjust for multiple comparisons, and statistical significance was defined as q < 0.05 (18).

Normality of continuous variables was assessed using the Shapiro–Wilk test. Because DNMT1, DNMT3a, folate, vitamin B6, and total homocysteine showed non-normal distributions, the Mann–Whitney U test was used for comparisons between groups.

We additionally performed an exploratory logistic regression analysis with schizophrenia diagnosis (0 = control, 1 = schizophrenia) as the dependent variable. Predictors included DNMT1 and DNMT3a expression levels, folate, vitamin B6, total homocysteine, age, and sex. Continuous predictors were standardized (per 1 SD). Model performance was summarized using the area under the ROC curve (AUC) and Nagelkerke R².

## Results

### Comparison of DNMT mRNA levels in schizophrenia patients and controls

**Figure 1:**
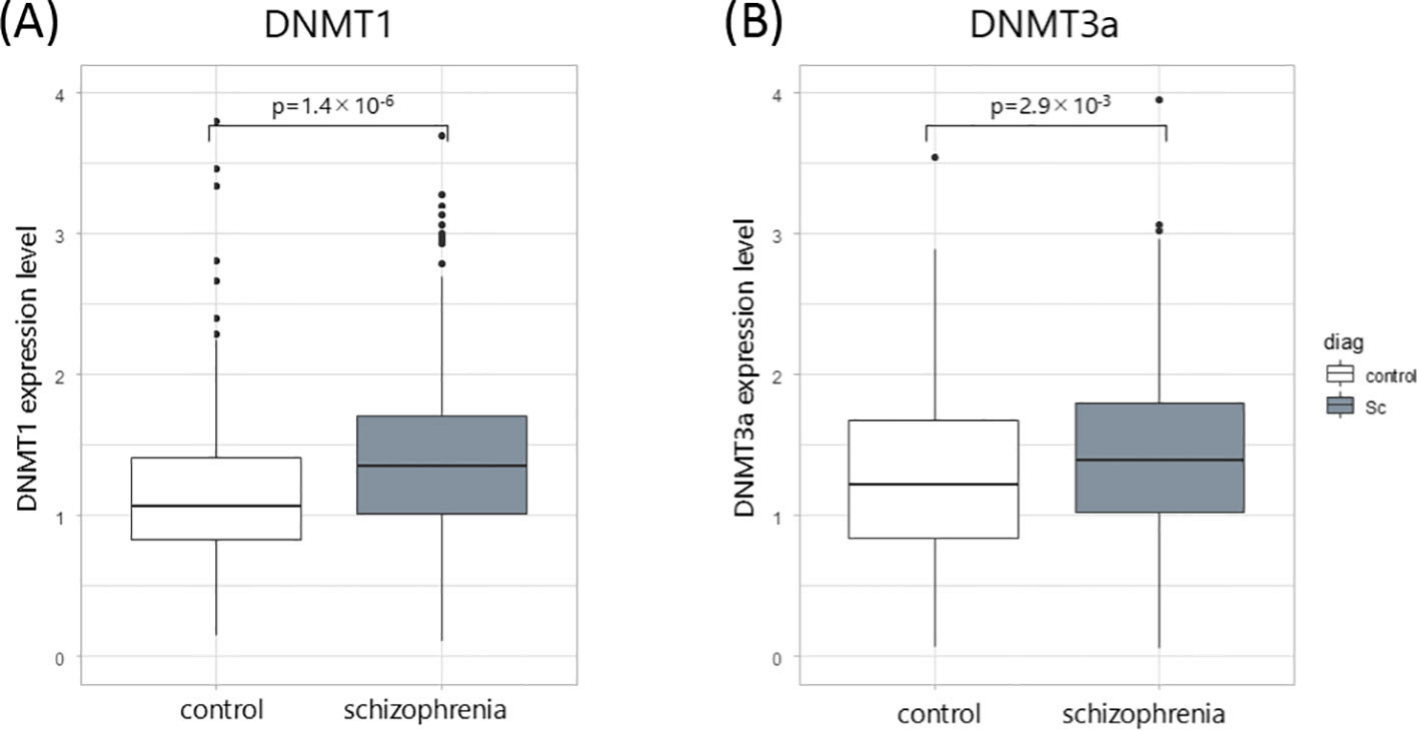
DNMT gene expressions. **(A)** Relative expression levels of DNMT1 in peripheral leukocytes from patients with chronic schizophrenia (n = 215) and age- and sex-matched healthy controls (n = 210). **(B)** Relative expression levels of DNMT3a in the same groups. Expression levels were quantified by quantitative real-time PCR and normalized to ACTB expression using the ΔΔCt method. Statistical comparisons were performed using the Mann–Whitney U test.

Expression levels of DNMT1 and DNMT3a in peripheral leukocytes were significantly higher in patients with chronic schizophrenia than in controls (p = 1.4 × 10–^6^ and 2.9 × 10^-3^, respectively) (**Figure 1**).

### Comparison of OCM-related factors in schizophrenia patients and controls

Serum folate and pyridoxal levels were significantly lower in patients with chronic schizophrenia than in controls (p = 2.0 × 10–^27^ and 3.1 × 10^-32^, respectively). Plasma total homocysteine (tHcy) levels were significantly higher in patients with chronic schizophrenia than in controls (p = 6.2 × 10^-18^).

### Relationship between DNMT expression and OCM metabolites

**Figure 2:**
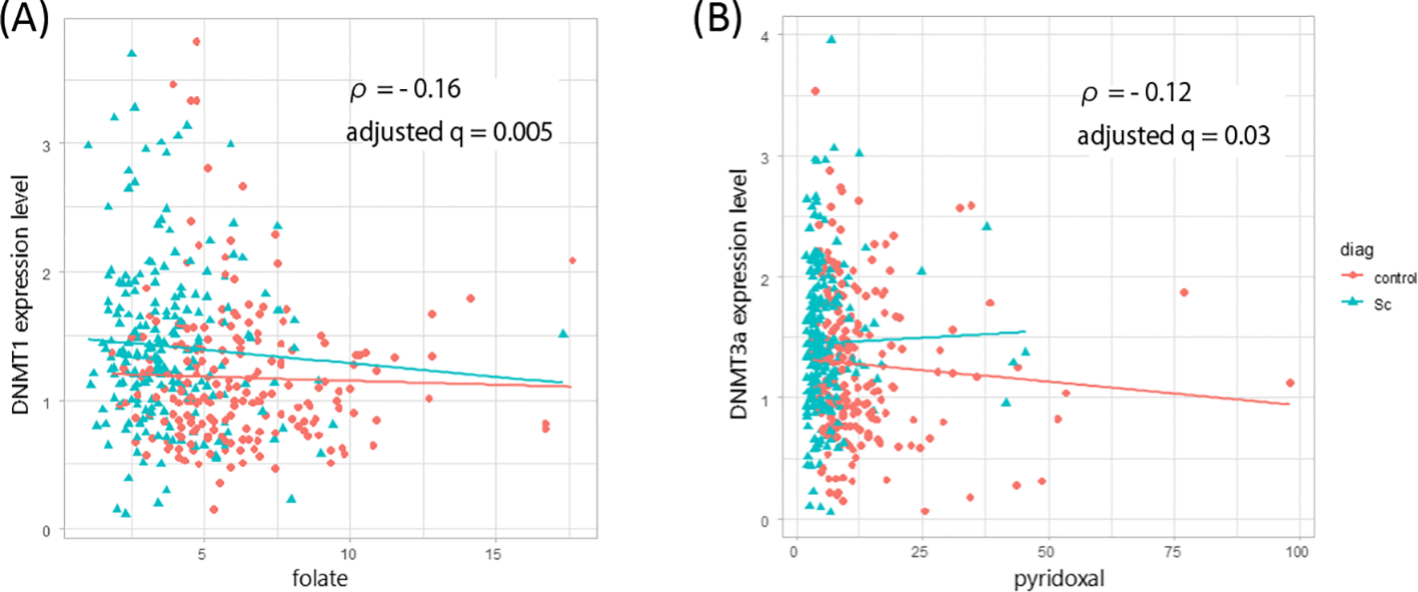
Correlations between DNMT gene expression levels and one-carbon metabolism–related factors. **(A)** Scatter plot showing the correlation between DNMT1 mRNA expression levels and serum folate concentration in the combined cohort (patients with schizophrenia, n = 215; controls, n = 210). **(B)** Scatter plot showing the correlation between DNMT3a mRNA expression levels and serum vitamin B6 (pyridoxal) concentration in the combined cohort. Statistical analysis was performed using Spearman’s correlation analysis. Solid lines indicate linear regression trends. ρ, Spearman’s correlation coefficient; adjusted q, false discovery rate–adjusted p-value.

The relationship between DNMT1 expression and OCM-related metabolites (folate, pyridoxal, and tHcy) was examined. DNMT1 mRNA expression was not significantly associated with either the schizophrenia group (N = 215) or the healthy group (N = 210) (adjusted q > 0.05). In the combined group (N = 425), a weak negative correlation was observed between DNMT1 mRNA expression and serum folate levels (ρ = −0.16, adjusted q = 5.0 × 10^-3^) (**Figure 2**). Similarly, DNMT3a expression was not significantly associated with any metabolite in either the schizophrenia or the control groups (adjusted q > 0.05). A weak negative correlation between DNMT3a mRNA levels and serum vitamin B6 was observed only in the combined groups (ρ = −0.12, adjusted q = 0.03) (**Figure 2**). In patients with schizophrenia, no significant correlations were observed between chlorpromazine-equivalent antipsychotic dose and DNMT1 (ρ = 0.06, p = 0.34) or DNMT3a expression levels (ρ = 0.03, p = 0.71).

Exploratory multivariable logistic regression showed that DNMT1 expression was independently associated with schizophrenia (OR = 1.41, 95% CI = 1.06–1.91, p = 0.020), whereas DNMT3a expression was not significant (p = 0.857). Lower folate (OR = 0.301, 95% CI = 0.200–0.439, p < 2×10^-9^) and lower vitamin B6 (OR = 0.253, 95% CI = 0.146–0.406, p < 2×10^-7^), as well as higher total homocysteine (OR = 2.13, 95% CI = 1.22–4.28, p = 0.019), were strong predictors of schizophrenia. The model demonstrated excellent discrimination (AUC = 0.88) and satisfactory explanatory power (Nagelkerke R² = 0.48).

## Discussion

This study demonstrated that DNMT1 and DNMT3a gene expression levels were elevated in the peripheral blood of patients with schizophrenia compared to those in healthy controls. These findings are consistent with previous studies examining peripheral blood and postmortem brain tissue (12–16). Elevated expression of DNMT1, which is involved in maintenance methylation, and DNMT3a, which mediates *de novo* methylation, may be associated with the hypermethylation observed in both peripheral blood and postmortem brain tissue in schizophrenia (6, 7, 19).

Multiple studies have indicated that DNMT1 and DNMT3a are involved in neurodevelopment and psychiatric disorders. For example, DNMT1 facilitates the migration of interneurons originating from the preoptic area to the cerebral cortex during brain development, thereby influencing the development of inhibitory interneurons (20). Experiments using Tet/Tet mouse embryonic stem cell lines suggested that both overexpression and absence of DNMT1 can adversely affect neural marker levels and impair neural cell differentiation (21). Genetic variants of DNMT1 have also been associated with specific symptom scores on the Positive and Negative Syndrome Scale (PANSS) (22). Noguchi et al. reported that deletion of DNMT1 from neural stem cells in adult mice not only7 impaired neurogenesis but also increased inflammatory features in the brains of adult mice, leading to anxiety-like behavior (23). These findings suggest that abnormal DNMT1 expression may be associated with neurodevelopmental abnormalities and schizophrenia. Kamei et al. reported that DNMT3a was upregulated in the adipose tissue of obese mice, and transgenic mice overexpressing DNMT3a exhibited increased expression of inflammatory cytokines induced by a high-fat diet (24). This finding suggests that abnormal DNMT3a expression may be associated with inflammation in schizophrenia.

We also observed significant negative correlations between serum folate concentration and DNMT1 gene expression and between serum vitamin B6 concentration and DNMT3a gene expression in the combined group. The exploratory logistic regression analysis indicated that DNMT1 expression, but not DNMT3a expression, was independently associated with schizophrenia after adjustment for one-carbon metabolites, age, and sex. The strong effects of folate, vitamin B6, and homocysteine suggest that general metabolic influences within the one-carbon pathway may be related to these associations. The logistic model showed excellent discrimination (AUC = 0.88) and good explanatory power (Nagelkerke R² = 0.48), supporting the robustness of these findings. To our knowledge, this is the first study to examine the association between DNMT gene expression and OCM-related factors. In our previous studies, we demonstrated an inverse correlation between serum folate and plasma total homocysteine, as well as a weak positive correlation between serum folate and vitamin B6 in the combined cohort (3–5). These findings suggest that interactions exist within the OCM pathway and highlight the need to further investigate this interaction framework to clarify the OCM abnormality hypothesis in schizophrenia.

Alterations in one-carbon metabolism have also been reported in other psychiatric disorders. A meta-analysis demonstrated that polymorphisms in the MTHFR gene are associated not only with schizophrenia but also with major depressive disorder (25). Furthermore, abnormalities in folate, vitamin B levels, and homocysteine metabolism have been described in bipolar disorder and obsessive–compulsive disorder (26–28). Together, these findings suggest that dysregulation of one-carbon metabolism may represent a transdiagnostic biological vulnerability rather than a mechanism specific to schizophrenia.

This study has certain limitations. Data on height and weight were not available for some patients with chronic schizophrenia; therefore, these participants were excluded from related analysis. Additionally, information on disease duration and hospitalization was not available; therefore, the association between the time course after disease onset and DNMT gene expression levels could not be examined. General clinical tests were not performed to exclude participants with physical comorbidities. In addition, the control participants were recruited from hospital staff and did not undergo a structured psychiatric interview such as the Structured Clinical Interview for DSM disorders. Although none reported a history of psychiatric illness, the presence of undiagnosed psychiatric conditions cannot be completely excluded, which may have introduced a potential bias. Furthermore, the relationship between DNMT gene expression and OCM-related factors, as well as its relationship with clinical symptoms, could not be examined because psychiatric symptoms were not assessed during blood collection. Although antipsychotic medications are known to influence epigenetic mechanisms, our exploratory analyses did not show significant associations between chlorpromazine-equivalent antipsychotic dose and DNMT1 or DNMT3a expression levels. Nevertheless, the potential effects of long-term medication exposure cannot be completely excluded. Finally, this study employed a cross-sectional design, so the causal relationships between observed variables cannot be inferred.

## Conclusion

DNMT1 and DNMT3a gene expression levels were significantly elevated in the peripheral blood of Japanese patients with chronic schizophrenia compared with healthy controls. Additionally, DNMT1 expression showed a weak negative correlation with serum folate, and DNMT3a expression was weakly negatively correlated with serum pyridoxal in the combined cohort. These findings contribute to the understanding of methylation and OCM abnormalities in schizophrenia.

## Data Availability

The datasets presented in this article are not readily available due to ethical and privacy restrictions. Requests to access the datasets should be directed to the corresponding author.
